# Un cas de nœud iléo-sigmoïdien chez une femme en post-partum

**DOI:** 10.11604/pamj.2019.32.106.13542

**Published:** 2019-03-07

**Authors:** Hind Boukhalit, Ouijdane Zamani, Laila Jroundi

**Affiliations:** 1 Université Mohamed V, Service de Radiologie des Urgences, Hôpital Avicenne, 10000, Rabat, Maroc

**Keywords:** Occlusion, nœud iléo-sigmoïdien, post-partum, Occlusion, ileosigmoid knotting, postpartum

## Abstract

Le nœud iléo-sigmoïdien est une cause rare d'occlusion intestinale. Nous rapportons un cas de nœud iléo-sigmoïdien survenu chez une femme en post-partum. Le diagnostic a été évoqué sur les données du scanner et confirmé en peropératoire.

## Abstract (en)

Ileosigmoid knotting is a rare cause of intestinal obstruction. We report a case of ileosigmoid knotting occurred during the postpartum period. The diagnosis was made by means of CT scan data and confirmed intraoperatively.

## Introduction

Le nœud iléo-sigmoïdien est un double volvulus intéressant le sigmoïde et le grêle, il évolue rapidement vers la nécrose intestinale. Le diagnostic préopératoire est difficile, la connaissance de son mécanisme et la recherche de signes scannographiques caractéristiques permet un diagnostic précoce et ainsi une prise en charge adaptée.

## Patient et observation

**Figure 1:**
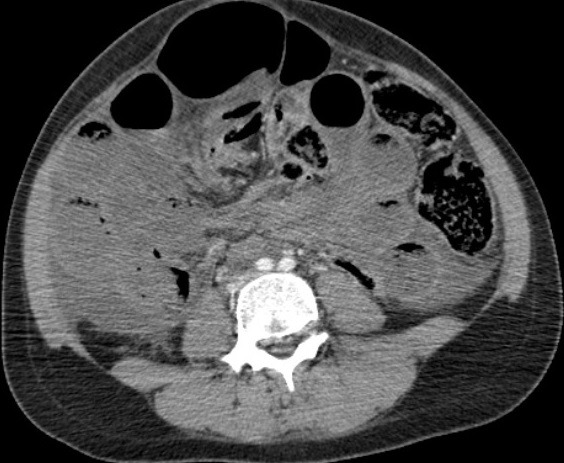
TDM abdominopelvienne en coupes axiales, avec injection de produit de contraste iodé, montrant une disposition radiaire pelvienne des anses grêles distendues avec un signe de tourbillon en regard, associée à la distension d’une boucle sigmoïdienne sans dilatation du côlon en amont

**Figure 2:**
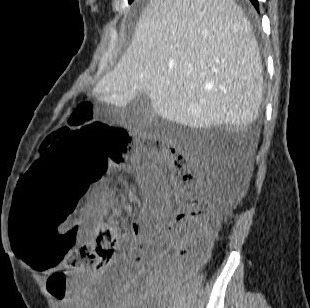
TDM abdominopelvienne en reconstructions sagittales, avec injection de produit de contraste iodé, en fenêtre parenchymateuse montrant la distension d’une boucle sigmoïdienne en amont d’une zone transitionnelle en bec

**Figure 3:**
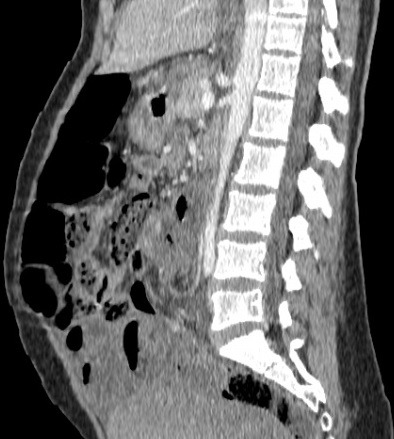
TDM abdominopelvienne en reconstructions sagittales, avec injection de produit de contraste iodé, en fenêtre parenchymateuse montrant la convergence des vaisseaux mésentériques vers les tours de spires

**Figure 4:**
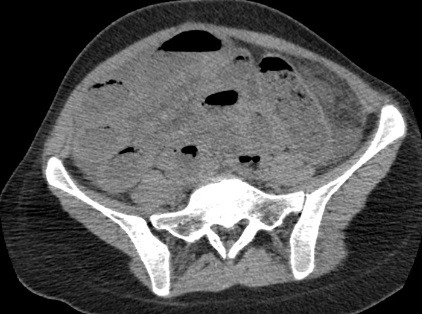
TDM abdominopelvienne en coupes axiales, sans injection de produit de contraste, montrant un aspect spontanément hyperdense des parois de l’iléon

**Figure 5:**
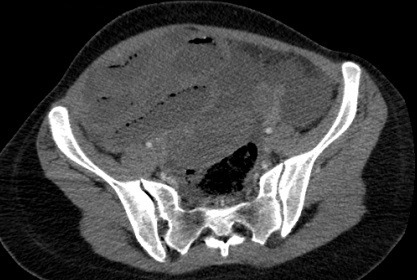
TDM abdominopelvienne en coupes axiales, avec injection de produit de contraste iodé, montrant un défaut de rehaussement avec pneumatose des parois de l’iléon

Il s’agit d’une jeune femme de 25 ans qui vient d’accoucher il y a 6 jours par voie basse. Admise au service urgences pour arrêt des matières et des gaz évoluant depuis 2 jours, associé à des coliques épisodiques initialement qui sont devenus permanentes. L'examen clinique trouve un abdomen distendu, un météorisme abdominal, une pâleur cutanéo-muqueuse diffuse avec une ampoule rectale vide. Le bilan biologique montre une anémie à 7g/dl d'hémoglobine. Un scanner abdominopelvien a été réalisé, il a montré d'une part une disposition radiaire des anses grêliques en situation pelvienne avec plusieurs zones de transition, associé à un signe de tourbillon en regard de L3 (Figure 1), prenant la partie distale de l'artère mésentérique supérieure, ces signes sont en faveur d'un volvulus de l'iléon. D'autre part il a montré la distension d'une boucle sigmoïdienne à plus de 70mm (Figure 1), en amont d'une zone transitionnelle en bec (Figure 2) sans dilatation du colon en amont, associée à la convergence des vaisseaux mésentériques inférieurs vers les tours de spires sus décrits (Figure 3), cet aspect est en faveur d'un volvulus sigmoïdien, on note l'absence de dilatation du colon en amont (Figure 1). L'aspect spontanément hyperdense (Figure 4) avec défaut de rehaussement et pneumatose des parois de l'iléon (Figure 5), associé à l'épanchement péritonéal (Figure 1, Figure 2, Figure 4, Figure 5) est compatible avec la souffrance ischémique digestive. Cet aspect radiologique est en faveur d'une double occlusion par un double volvulus iléal et sigmoïdien. La patiente a été opérée en urgence, confirmant le volvulus du sigmoïde sur un nœud réalisé par l'iléon volvulé, c'est le nœud iléo-sigmoïdien, avec nécrose iléale étendue, la patiente a bénéficié d'une résection iléale et d'une stomie.

## Discussion

Le volvulus iléo-sigmoïdien, ou nœud iléo-sigmoïdien (NIS) est un « nœud» créé par un volvulus du côlon sigmoïde et de l'intestin grêle, plus particulièrement l'iléon. Il a été décrit pour la première fois par Parker en 1845 [1]. Le NIS est une entité rare, représente 7,6% de l'ensemble des volvulus du sigmoïde en France [2]. Plusieurs facteurs ont été incriminés pour expliquer cette pathologie, Atamanalp *et al*. [3] ont évoqué des prédispositions anatomiques, un intestin grêle hypermobile par un méso trop long et une racine courte peut s'enrouler au pied du côlon sigmoïde. Un deuxième facteur est d'ordre alimentaire, la réplétion rapide du jéjunum chez les patients qui mangent un seul repas par jour favoriserait sa torsion autour de l'iléon vide, emportant ainsi la boucle sigmoïdienne [4,5]. Alver *et al* [2] décrivent 4 types de mécanismes de formation du NIS, selon le segment digestif actif responsable de la torsion, dans le type I l'iléon est le segment actif s'enroulant autour du sigmoïde passif, le type II résulte de la torsion sigmoïdienne active qui attire le grêle passif, dans le type III exceptionnel c'est la jonction iléo-caecale qui s'enroule autour de la boucle sigmoïdienne, tandis que dans le type IV indéterminé il n'est pas possible de différencier les deux segments. Le NIS entraine une occlusion intestinale complexe par double strangulation des vaisseaux mésentériques à destinée des anses grêles et du sigmoïde, ce mécanisme aboutit à une nécrose ischémique rapide des deux segments volvulés [6]. Le diagnostic préopératoire est difficile en raison de sa rareté et d'une atypie clinico-radiologique, il est possible dans moins de 20% des cas [1, 7, 8].

Le syndrome occlusif clinique est marqué par les douleurs abdominales aigues initialement localisées, puis permanentes et généralisées, un tableau d'hypovolémie est évocateur dans 56% des cas [7-9]. La radiographie de l'abdomen sans préparation peut montrer occasionnellement les caractéristiques d'une double occlusion à boucle fermée avec des niveaux hydro-aériques sigmoïdiens dans le quadrant supérieur droit, et d'autres de type grêlique pouvant être latéralisées à gauche [2, 4, 10], le plus souvent elle montre un volvulus sigmoïdien ou une occlusion grêlique isolée. Le scanner abdominopelvien confirme l'occlusion sigmoïdienne et iléale, permet de chercher outre les signes de l'ischémie intestinale, des signes caractéristiques du NIS, le tour de spire est plus volumineux que dans un volvulus isolé du sigmoïde, portant les vaisseaux mésentériques supérieurs et inférieurs [10], cet aspect pourrait s'expliquer par le fait que les deux volvulus se superposent [6]. La rétention des matières dans le colon proximal non distendu et la disposition radiaire des anses grêliques orientent le diagnostic selon Hashimoto *et al* [11]. L'association de la déviation médiale du colon descendant et du mesocœcum, avec aspect effilé et pointu de leurs bord internes, convergeant vers des tours de spires est très caractéristique du nœud iléo-caecal [3, 10, 12], cet aspect est dû d'une part à l'attraction du péritoine pariéto-colique gauche et du mésocoecum vers le centre du NIS, et d'autre part à l'effet de masse des anses grêles dilatées [10]. Le NIS est associé à une mortalité élevée nécessitant un diagnostic précoce, une prise en charge chirurgicale adaptée et rapide, le traitement est basé sur la résection-anastomose ou la colostomie si les segments intestinaux sont nécrosés, la détorsion et la sigmoïdopexie est recommandée dans les rares cas où il n'y a pas de nécrose [12].

## Conclusion

Le nœud iléo-sigmoïdien est une cause rare d'occlusion intestinale, de diagnostic difficile, l'évolution se fait rapidement vers la nécrose digestive. La découverte d'un aspect de double volvulus au scanner incite à une intervention chirurgicale urgente. La bonne compréhension du mécanisme et le diagnostic radiologique préopératoire permettent de diminuer la morbi-mortalité.

## Conflits d’intérêts

Les auteurs ne déclarent aucun conflit d'intérêts.
